# *Pseudomonas viridiflava*, a Multi Host Plant Pathogen with Significant Genetic Variation at the Molecular Level

**DOI:** 10.1371/journal.pone.0036090

**Published:** 2012-04-27

**Authors:** Panagiotis F. Sarris, Emmanouil A. Trantas, Evaggelia Mpalantinaki, Filippos Ververidis, Dimitrios E. Goumas

**Affiliations:** Department of Plant Sciences, School of Agricultural Technology, Technological Educational Institute of Crete, Heraklion, Greece; Nanjing Agricultural University, China

## Abstract

The pectinolytic species *Pseudomonas viridiflava* has a wide host range among plants, causing foliar and stem necrotic lesions and basal stem and root rots. However, little is known about the molecular evolution of this species. In this study we investigated the intraspecies genetic variation of *P. viridiflava* amongst local (Cretan), as well as international isolates of the pathogen. The genetic and phenotypic variability were investigated by molecular fingerprinting (rep-PCR) and partial sequencing of three housekeeping genes (*gyrB*, *rpoD* and *rpoB*), and by biochemical and pathogenicity profiling. The biochemical tests and pathogenicity profiling did not reveal any variability among the isolates studied. However, the molecular fingerprinting patterns and housekeeping gene sequences clearly differentiated them. In a broader phylogenetic comparison of housekeeping gene sequences deposited in GenBank, significant genetic variability at the molecular level was found between isolates of *P. viridiflava* originated from different host species as well as among isolates from the same host. Our results provide a basis for more comprehensive understanding of the biology, sources and shifts in genetic diversity and evolution of *P. viridiflava* populations and should support the development of molecular identification tools and epidemiological studies in diseases caused by this species.

## Introduction

*Pseudomonas* species are ubiquitous bacteria endowed with metabolism that enables them to dwell in a large variety of environmental niches. Various *Pseudomonas* species are important as pathogens of animals, insects and plants [1]–[3]. The molecular taxonomic criteria for the genus *Pseudomonas* have been revised along with the progress in bacterial taxonomy. However, due to the inability of DNA-DNA hybridization and 16S rDNA-based methods to reveal intraspecies variability, Yamamoto and colleagues suggested that a phylogenetic analysis using the nucleotide sequences of the housekeeping genes for the beta subunit of the DNA gyrase (*gyrB*) and σ^70^ RpoD protein subunit of RNA polymerase (*rpoD*), which evolve much faster than rDNAs [1], provide the higher resolution necessary for intraspecies variation analysis than 16S rDNA sequences [4].

Traditionally, the phytopathogenic oxidase-negative fluorescent Pseudomonads have been grouped into two species, *Pseudomonas syringae* and *Pseudomonas viridiflava* [5]. The LOPAT determinative tests (L: levan production; O: oxidase production; P: pectinolytic activity; A: arginine dihydrolase production; and T: tobacco hypersensitivity) are the most widely used protocol for the differentiation of plant pathogenic Pseudomonads [6], [7].

The pectinolytic species *P. viridiflava* (Burkholder) Dowson, [8], [9] has a wide range of hosts causing necrotic leaf and stem lesions and basal stem and root rots. It was originally isolated from the dwarf or runner bean, in Switzerland (reference strain *P. viridiflava* ATCC13223). However, based on 16S rDNA analysis, *P. viridiflava* had been placed previously in the *P. syringae* group [10]. Likewise, following ribotypical analysis, strains of *Pseudomonas syringae* pv. *ribicola* (infects *Ribes aureum*) and *Pseudomonas syringae* pv. *primulae* (infects *Primula* species) were also incorporated into the *P. viridiflava* species [11].

*P. viridiflava* is a multihost pathogen causing severe damages to tomato (*Solanum lycopersicum*) [12], [13], melon (*Cucumis melo*) [14], [15], blite (*Amaranthus blitum*), chrysanthemum (*Chrysanthemum morifolium*), eggplant (*Solanum melongena*) [15], and the model plant species *Arabidopsis thaliana* [16]. Typical symptoms of *P. viridiflava* infection in tomato are a general wilting and yellowing of the plants and brown-black spots developing at the pruning sites of the stem. In the inner part of the stem, pith and vascular tissues display brown discolouration and soft rot often develops. It is a significant pathogen in the eastern Mediterranean region and Aegean islands in particular, representing 12% and 50%, respectively, of the *Pseudomonas* species causing stem necrosis [17], [18].

The aim of the present study was to evaluate the genetic variation among local and global isolates of *P. viridiflava*. Several strains from laboratory collections and new isolates from several plant species were studied by a) biochemical markers, b) pathogenicity profiling, c) molecular fingerprinting and d) partial sequencing of the housekeeping genes *gyrB* (DNA gyrase beta subunit), *rpoD* (RNA polymerase σ^70^ subunit) and *rpoB* (RNA polymerase beta subunit) which have been used assignatures for bacterial identification, as well as loci for phylogenetic analysis [19]. To our knowledge, this is the first report worldwide of *P. viridiflava* being a pathogen on *Acanthus mollis* and capitulum bracts of *Cynara scolymus* L.

## Results

### Biochemical Profiling

On the basis of their colony morphology, physiological, biochemical, and pathological characteristics, representative isolates of *Pseudomonas* spp. were identified as *P. viridiflava* based on the determinative schemes as proposed previously by various researchers [7], [20], [21]. Eighteen local (Crete, Greece) isolates were chosen (Table 1) for further characterization, using the LOPAT tests, together with the reference strain *P. viridiflava* NCPPB1249 and other fluorescent *Pseudomonas* species (Tables 1 and 2). Supplementary rapid identification of isolates was achieved by using the pattern of fluorescence on single carbon source media [Sucrose:(−), Erythritol:(+) and DL-Lactate:(+)] as described in [22]. All tested isolates gave identical results in these tests as well as in the biochemical profiling to the *P. viridiflava* reference strain and were clearly differentiated from the other fluorescent *Pseudomonas* species (Table 3 and Table S2). A unique exception was seen in the L(+) Tartrate utilization test in which only the tomato isolates tested positive, in contrast to the type strain (Table 3 and Table S2) and the local isolates from other hosts. Thus, the results of the biochemical identification tests did not indicate any variability among the local *P. viridiflava* isolates examined, with the above mentioned exception.

**Table 1:** Bacterial strains used in this study for biochemical characterization and pathogenicity tests.

	Code	Host	Disease/symptoms	Location	Origin
*Pseudomonas viridiflava*	PV271	*Apium graveolens* L.	Celery leaf blight	Heraklion, Crete	This study
	PV272	*Apium graveolens* L.	Celery leaf blight	Heraklion, Crete	This study
	PV272a	*Apium graveolens* L.	Celery leaf blight	Heraklion, Crete	This study
	PV273	*Apium graveolens* L.	Celery leaf blight	Heraklion, Crete	This study
	PV273a	*Apium graveolens* L.	Celery leaf blight	Heraklion, Crete	This study
	PV274	*Apium graveolens* L.	Celery leaf blight	Heraklion, Crete	This study
	PV276	*Apium graveolens* L.	Celery leaf blight	Heraklion, Crete	This study
	PV612	*Cucumis melo* cv Naudin	Cantaloupe leaf spot/necrosis	Tympaki, Crete	[13]
	PV527	*Amaranthus blitum* L.	Blite (purple amaranth) leaf spot	St. Pelagia, Crete	[13]
	PV3005	*Solanum melongena* L.	Eggplant leaf spot	Ierapetra, Crete	[13]
	PV3006	*Solanum melongena* L.	Eggplant leaf spot	Ierapetra, Crete	[13]
	PV570	*Acanthus mollis* L.	Bear's Breeches leaf blight	Heraklion, Crete	This study
	PV574a	*Acanthus mollis* L.	Bear's Breeches leaf blight	Heraklion, Crete	This study
	TKK615	*Solanum lycopersicum*	Tomato spot on fruit	Antiskari, Crete	[15]
	PV441	*Solanum lycopersicum*	Tomato stem soft rot; pith necrosis	Tympaki, Crete	[13]
	PV442	*Solanum lycopersicum*	Tomato stem soft rot; pith necrosis	Tympaki, Crete	[15]
	PV608	*Cynara scolymus* L.	Artichoke bracts leave lesions/necrosis	Heraklion, Crete	This study
	PV609	*Cynara scolymus* L.	Artichoke bracts leave lesions/necrosis	Heraklion, Crete	This study
	NCPPB1249	*Chrysanthemummorifolium*	Stem soft rot	United Kingdom (1962)	[15]
*P. savastanoi* pv. *savastanoi*	Ps.sav1	*Olea europaea*	Olive knot disease	Heraklion, Crete	This study
	Ps.sav4	*Olea europaea*	Olive knot disease	Heraklion, Crete	This study
	Ps.sav5	*Olea europaea*	Olive knot disease	Heraklion, Crete	This study
*P. syringae* pv. t*omato*	Pst1	*Solanum lycopersicum*	Tomato bacterial speck disease	Tympaki, Crete	This study
	Pst2	*Solanum lycopersicum*	Tomato bacterial speck disease	Tympaki, Crete	This study
	Pst3	*Solanum lycopersicum*	Tomato bacterial speck disease	Tympaki, Crete	This study
*P. syringae* pv. l*achrymans*	Psl110	*Cucumis sativus*	Angular leaf spot of Cucurbits	Ierapetra Crete	[13]
	Psl119	*Cucumis sativus*	Angular leaf spot of Cucurbits	Ierapetra Crete	[13]
	Psl102	*Cucumis melo*	Angular leaf spot of Cucurbits	Lasithi Crete	[13]
*P. syringae* pv. *syringae*	Pss11	*Citrus lemon*	Citrus blast disease, black pit	Fodele Crete	[13]
	NCPPB2778	*Pyrus communis*	Pear blossom blast and canker	France (1965)	[13]

**Table 2:** LOPAT tests of eighteen local (Crete, Greece) *P. viridiflava* isolates along with *P. viridiflava* reference strain NCPPB1249 and other pseudomonads.

Species	Strain No	Levan	Oxidase	Potato rot	Arginine	Tobacco (HR)	Fluorescence pigment
*Pseudomonas viridiflava*	PV271	−	-	+	-	+	+Blue
	PV272	−	-	+	-	+	+Blue
	PV272α	−	-	+	-	+	+Blue
	PV273	−	-	+	-	+	+Blue
	PV273α	−	-	+	-	+	+Blue
	PV274	−	-	+	-	+	+Blue
	PV276	−	-	+	-	+	+Blue
	PV612	−	-	+	-	+	+Blue
	PV527	−	-	+	-	+	+Blue
	PV3005	−	-	+	-	+	+Blue
	PV3006	−	-	+	-	+	+Blue
	PV570	−	-	+	-	+	+Blue
	PV574a	−	-	+	-	+	+Blue
	TKK615	−	-	+	-	+	+Blue
	PV441	−	-	+	-	+	+Blue
	PV442	−	-	+	-	+	+Blue
	PV608	−	-	+	-	+	+Blue
	PV609	−	-	+	-	+	+Blue
	NCPPB1249	−	-	+	-	+	+Blue
*P. syringae*	All strains	+	-	−	-	+	Green-Blue
*P. savastanoi*	All strains	+	-	−	-	+	Green

**Table 3:** Comparison of *P. viridiflava* local isolates from different hosts found in the island of Crete and other fluorescent *Pseudomonas* species (Table 1) used in differential nutritional, biochemical and other tests.

	*Solanum lycopersicum*	*Solanum melongena*	*Apium graveolens*	*Amaranthus blitum*	*Cynara scolymus* & *Acanthus mollis*	*Cucumis melo*	*P. viridiflava* NCPPB1249	Other *Pseudomonas* species			
								*syringae* pv. *lachrymans*	*savastanoi* pv. *savastanoi*	*syringae* pv. *syringae*	*syringae* pv. *tomato*
**Tests**											
Levan	−	−	−	−	−	−	−	+	+	+	+
Oxidase	−	−	−	−	−	−	−	−	−	−	−
Potato rot	+	+	+	+	+	+	+	−	−	−	−
Arginine dihydrolase	−	−	−	−	−	−	−	−	−	−	−
Hypersensitivity	+	+	+	+	+	+	+	+	+	+	+
Nitrate reduction	−	−	−	−	−	−	−	−	−	−	−
Fluorescent pigment	+	+	+	+	+	+	+	+	+	+	+
Gelatin hydrolysis	+	+	+	+	+	+	+	−	+	+	−
Pectate gel pitting^2^	+	+	+	+	+	+	+	NT	−	−	NT
Lipases	+	+	+	+	+	+	+	NT	NT	+	NT
2-Ketogluconate	−	−	−	−	−	−	−	−	NT	−	−

(+) = positive; (−) = negative; NT = not available. Information for additional biochemical experiments can be found in supplementary Table S2.

### Pathogenic Profiling and Disease Symptomatology

Similarly to the biochemical profiling, all *P. viridiflava* local isolates examined had identical pathogenicity profiles when tested on a series of experimental host species (Table S1). Successful inoculations were made on tomato, eggplant, blite, melon, celery, artichoke, acanthus and chrysanthemum under greenhouse conditions. In each host, the symptoms induced by a strain were similar to those caused by each *P. viridiflava* isolate on its natural host (Figure S1). In other words, each isolate induced the same disease symptoms independently of host of origin. On tomato, eggplant, blite, melon, celery and acanthus leaves, the disease started as a water-soaked spot which developed in 3–4 days into small or larger irregular lesions, usually with chlorotic halos. The centre of the lesions later became dry and tan to black in colour. Later the lesions usually coalesced and leaves appeared blighted. Tomato and chrysanthemum plants that were stab-inoculated into the stem developed yellowing in the lower leaves, wilting, and a yellow to brown discoloured pith within 6–10 days. The stem often became hollow and split with bacterial slime exudating. On artichoke, the disease started as water-soaked and dark-green spots on the capitulum bracts. Infected leaves developed sunken and elongated necrotic lesions with a brown to black centre surrounded by thin water-soaked halos along with large dark red-brown margins.

Re-isolations made from the artificially infected plants yielded pure cultures that were confirmed as *P. viridiflava* by LOPAT tests. All local isolates of *P. viridiflava* regardless of their original hosts (Table 1) caused rust-coloured lesions within 48 h on excised snap bean pods, induced soft rots on pear and did not produce the deep black necrotic pit symptoms on detached lemon fruits [13], [15] (Figure S2). The results of the pathogenicity profiling also did not reveal any variability among the *P. viridiflava* strains under study on the plants used for experimental inoculations. However, a validation of the present results against a broader host sampling scheme and detailed phytopathological characterization (e.g. estimation of pathotypes, race specificity, etc.), may provide more relevant information about the intraspecific level of variation of *P. viridiflava* isolates studied.

### Molecular Fingerprinting

To further investigate inter-strain variability of the local *P. viridiflava* isolates (Table 1), we utilized BOX- (mosaic repetitive sequences of dyad symmetry within intergenic regions), and ERIC- (Enterobacterial Repetitive Intergenic Consensus) like DNA sequences corresponding to conserved repetitive bacterial motifs (collectively known as rep-PCR) to generate genomic fingerprints [23]–[25]. Rep-PCR fingerprinting is a useful and reliable technique to assess bacterial diversity at the species, subspecies, or even isolate level; its applications to environmental microbiology have been reviewed [26]. This method has high capacity to snap-shot the whole genome, showing greater discriminatory power than PFGE (Pulsed-Field Gel Electrophoresis) and MLST (Multilocus Sequence Typing) [27], in comparison with various other phylogenetic methods in bacterial typing and phylogeny [27]–[32].

**Figure 1:**
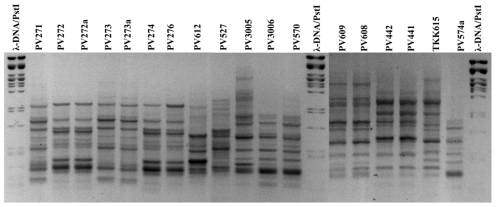
Agarose gel electrophoresis of BOX-PCR of 18 local *P. viridiflava* isolates. Agarose gel electrophoresis of BOX-PCR amplification products from genomic DNA of 18 local *P. viridiflava* isolates. The molecular size marker is λ phage DNA digested with the restriction endonuclease *Pst*I. The negative film filter was applied to the image of an ethidium bromide gel. Isolate codes are given over each lane.

**Figure 2:**
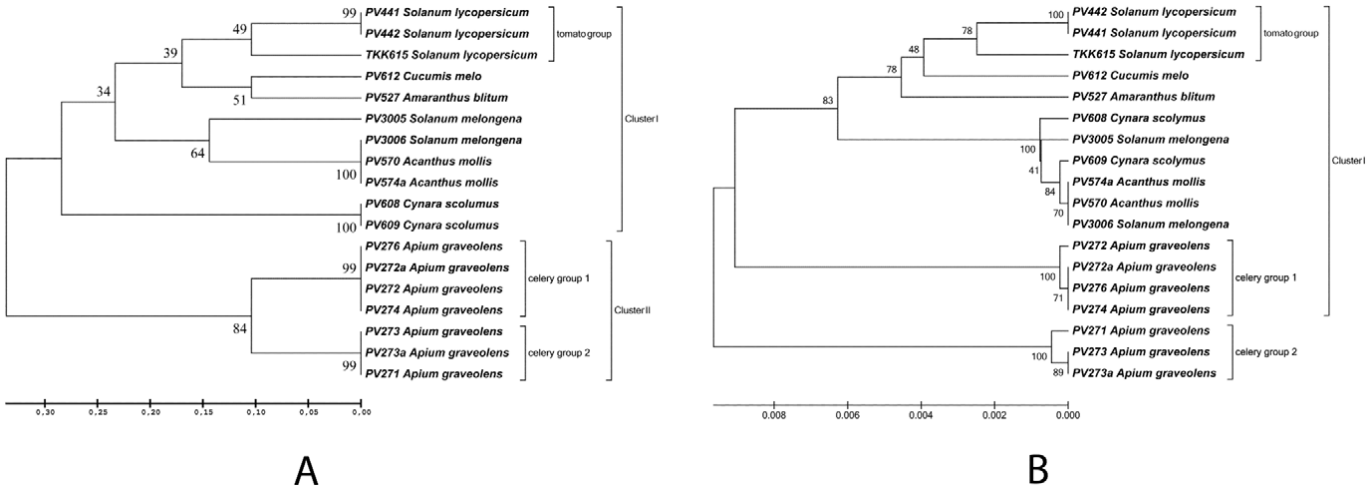
Phylogenetic trees of the local *P. viridiflava* isolates. The construction of the dendrograms was based on **A**: BOX- and ERIC-PCR fingerprints (rep-PCR) and **B**: the combined *gyrB, rpoD* and *rpoB* gene sequences. The plant hosts are given next to the code number (PVXXX, see Table 1) of each isolate. The evolutionary history was inferred using the UPGMA method. The consensus tree inferred from 1500 replicates is taken to represent the evolutionary history of the isolates analyzed. Branches corresponding to partitions reproduced in less than 50% bootstrap replicates are collapsed. The percentage of replicate trees in which the associated taxa clustered together in the bootstrap test is shown next to the branches. The tree is drawn to scale, with branch lengths in the same units as those of the evolutionary distances used to infer the phylogenetic tree. The evolutionary distances were computed using the Maximum Composite Likelihood method and are in the units of the number of base substitutions per site. All positions containing gaps and missing data were eliminated. There were a total of 2222 positions in the final dataset. Evolutionary analyses were conducted in MEGA5.

The rep-PCR amplifications on total DNA of the eighteen *P. viridiflava* strains showed 9–18 bands in the case of BOX-PCR (Figure 1), and 10–20 bands in the case of ERIC-PCR. A total of 16 discrete bands were scored in both fingerprinting methods, ranging in size from 0.15 kb to 2.6 kb. The data matrix showing presence or absence of the scored bands was analysed with the Jaccard's coefficient and a combined BOX and ERIC dendrogram [33]–[35] was created with UPGMA (Figure 2A). All isolates were clustered in two distinct major clusters. The first cluster (Figure 2A; cluster I) included isolates from tomato, eggplant, melon, blite, acanthus and artichoke while the second cluster (Figure 2A; cluster II) contained only the celery isolates (Figure 2A; celery groups 1 and 2).

The first cluster showed the greatest variability and was further divided into three sub-clusters which correlated with the host of origin (Figure 2A; Cluster I). One sub-cluster consisted of the tomato isolates (Figure 2A; tomato group) which were similar to the blite and melon isolates. The second sub-cluster consisted of the eggplant and acanthus isolates. In the third sub-cluster the isolates from artichoke were grouped. The analysis linked closely all strains isolated from the same host, indicating a common genetic base. More specifically, in the eggplant-acanthus sub-cluster, the isolates PV3006, PV570 and PV574a had identical fingerprinting profiles, while the strain PV3005, isolated from eggplant, was clearly differentiated. The tomato isolates PV441 and PV442 had the same fingerprint but were slightly different from the TKK615 isolate.

The second major rep-PCR cluster was also divided in two sub-clusters with a remarkably high bootstrap value (84%), indicating genetic variability among the isolates from the same host plant (celery). These results led us to conclude that BOX- and ERIC-PCR seem to be able to identify the genetic variability at the intra-species level among *P. viridiflava* isolates, with only few exceptions.

### Phylogeny based on *gyrB*, *rpoD* and *rpoB* gene sequences

Further analysis of inter-isolate variability was carried out on nucleotide sequences of three PCR-amplified housekeeping gene regions (*gyrB* 840 bp, *rpoD* 615 bp and *rpoB* 1250 bp; total sequence length 2705 bp) for the eighteen local *P. viridiflava* isolates (GenBank accession numbers are given in Table 4 and Table S3), using the Jaccard coefficient. The UPGMA trees generated gave a very good fit when checked by the Mantel test [27](0.94628, 0.97996 and 0.83253 for *gyrB, rpoD* and *rpoB*, respectively). Furthermore, the basic topologies were preserved in the generated trees (Figure S3A), with the *gyrB* sequence tree providing higher resolution in the final consensus tree obtained with the combined sequences of all three genes (Figure 2B).

**Table 4:** Local bacterial strains used in this study for *gyrB*, *rpoD* and *rpoB* phylogenetic analysis.

Strain No.	Host	*gyrB* GenBank No.	*rpoD* GenBank No.	*rpoB* GenBank No.	Origin
PV271	*Apium graveolens* L.	JN383377	JN383347	JQ267553	This study
PV272	*Apium graveolens* L.	JN383378	JN383348	JQ267548	This study
PV272a	*Apium graveolens* L.	JN383379	JN383349	JQ267555	This study
PV273	*Apium graveolens* L.	JN383380	JN383350	JQ267550	This study
PV273a	*Apium graveolens* L.	JN383381	JN383351	JQ267552	This study
PV274	*Apium graveolens* L.	JN383382	JN383352	JQ267557	This study
PV276	*Apium graveolens* L.	JN383365	JN383353	JQ267556	This study
PV612	*Cucumis melo* cv. Naudin	JN383366	JN383354	JQ267551	This study
PV527	*Amaranthus blitum* L.	JN383367	JN383355	JQ267560	This study
PV3005	*Solanum melongena* L.	JN383368	JN383356	JQ267559	This study
PV3006	*Solanum melongena* L.	JN383369	JN383357	JQ267561	This study
PV570	*Acanthus mollis* L.	JN383370	JN383358	JQ267558	This study
PV574a	*Acanthus mollis* L.	JN383371	JN383359	JQ267554	This study
TKK615	*Solanum lycopersicum*	JN383372	JN383360	JQ267549	This study
PV441	*Solanum lycopersicum*	JN383373	JN383361	JQ267544	This study
PV442	*Solanum lycopersicum*	JN383374	JN383362	JQ267545	This study
PV608	*Cynara scolymus* L.	JN383375	JN383363	JQ267546	This study
PV609	*Cynara scolymus* L.	JN383376	JN383364	JQ267547	This study

Information for other strains used, can be found in supplementary Table S3.

In general, the Jacquard's coefficient created the two major clusters seen with the BOX-ERIC tree and grouped the celery genotypes into two subgroups, although somewhat differently (Figure S3; celery groups 1 and 2). One subgroup consisted of the genotypes PV276, PV272a, PV272, PV274 and the second subgroup of the genotypes PV273a, PV273 and PV271. Nevertheless, only the *gyrB* phylogeny separated the celery sub-groups from the rest of the genotypes, linking them in a major cluster (Figure S3A; cluster II) which comprised only celery isolates. When the taxonomy was based on *rpoD* sequences (Figure S3B) the celery isolates were grouped in two distinct sub-clusters, the first (PV276, PV272a, PV272, PV274, celery group 1) being linked closer to isolates from eggplant, tomato, acanthus, artichoke and melon, and the second containing the celery isolates PV273a, PV273 and PV271 (celery group 2), was closer to the blite isolate and distantly linked to the rest of the *P. viridiflava* isolates. Another noticeable difference between the *gyrB* and *rpoD* trees was that in the *rpoD* tree the tomato isolate TKK615 did not group with the rest of tomato isolates (PV441 and PV442, Figure S3B), as was the case in the *gyrB* tree but was placed closer to eggplant, acanthus, and artichoke isolates (Figure S3A, B).

When the *rpoB* gene sequence was implemented, the constructed tree (Figure S3C) was much more similar to the *rpoD* tree rather than to the *gyrB* tree, preserving the general qualitative characteristics of the former. Only one celery sub-group was clearly created, which contained the isolates PV276, PV272a, PV272 and PV274, while the genotypes of the strains that belong to the second *gyrB* and *rpoD* celery sub-group were mixed with those of strains isolated from melon (PV612) tomato (PV441, PV442) and blite (PV527).

Theoretically, the influence of stochastic drift on the rate of evolution could be eliminated from the molecular phylogeny. Hence, these minor discrepancies in the above groupings may have their origin in such drift. If this was the case, the parallel use of all three genes in the analysis should give a more accurate estimate of the phylogeny [1]. Therefore, we forced the software to reckon phylogenetic analysis by combining the partial sequences of the *gyrB*, *rpoD* and *rpoB* genes. A UPGMA dendrogram was reconstructed and is presented in Figure 2B. The topology of the tree from the combined gene sequences follows the phylogeny of the *gyrB* and *rpoD* genes.

**Figure 3:**
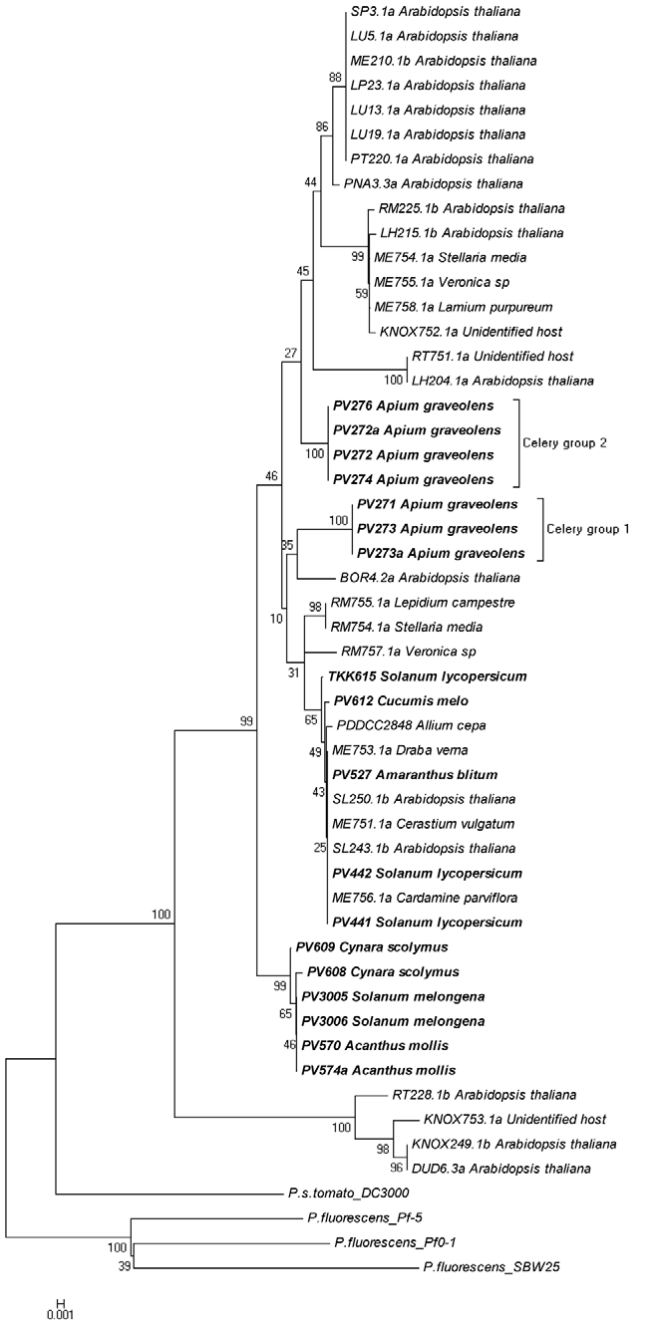
*P. viridiflava* phylogenetic tree, utilizing *gyrB* sequences determined in this study along with sequences obtained from GenBank. The evolutionary history was inferred using the Neighbor-Joining method. The bootstrap consensus tree inferred from 1500 replicates is taken to represent the evolutionary history of the taxa analyzed. Branches corresponding to partitions reproduced in less than 50% bootstrap replicates are collapsed. The percentage of replicate trees in which the associated taxa clustered together in the bootstrap test are shown next to the branches. The tree is drawn to scale, with branch lengths in the same units as those of the evolutionary distances used to infer the phylogenetic tree. The evolutionary distances were computed using the Maximum Composite Likelihood method and are in the units of the number of base substitutions per site. The analysis involved 52 nucleotide sequences. All positions containing gaps and missing data were eliminated. There were a total of 740 positions in the final dataset. Evolutionary analyses were conducted in MEGA5. The host plant species is presented next to the code number (e.g. PVXXX) of each isolate.

**Figure 4:**
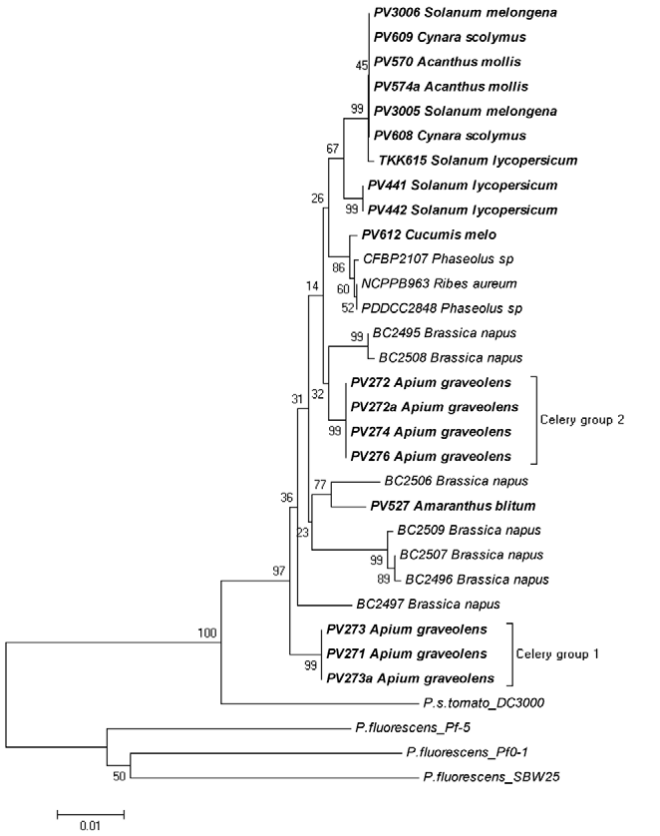
*P. viridiflava* phylogenetic trees, utilizing *rpoD* sequences along with sequences obtained from GenBank. The evolutionary history was inferred using the Neighbor-Joining method. Tree construction and evolutionary distances were carried out as described in the Figure 2 legend. The analysis involved 32 nucleotide sequences. All positions containing gaps and missing data were eliminated. There were a total of 513 positions in the final dataset. The methodology used for the evolutionary analysis, tree construction and other details are described in the Figure 3 legend.

**Figure 5:**
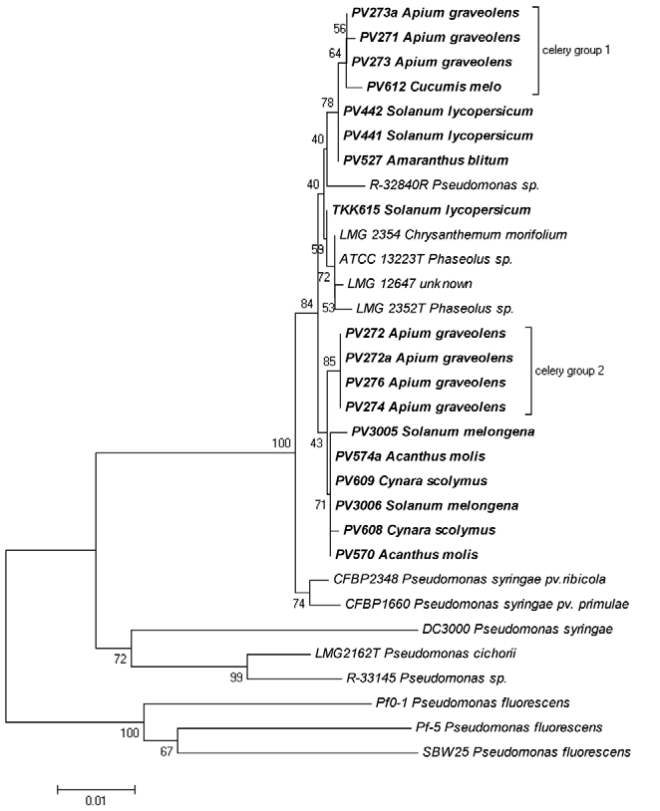
*P. viridiflava* phylogenetic trees, utilizing *rpoB* sequences along with sequences obtained from GenBank. The evolutionary history was inferred using the Neighbor-Joining method. Tree construction and evolutionary distances were carried out as described in the Figure 2 legend. The analysis involved 27 nucleotide sequences. All positions containing gaps and missing data were eliminated. There were a total of 741 positions in the final dataset. The methodology used for the evolutionary analysis, tree construction and other details are described in the Figure 3 legend.

The third step in our analysis was to examine the linkage between the local *P. viridiflava gyrB, rpoD* and *rpoB* gene sequences along with those deposited in GenBank (Tables 4, S3 and the resulting trees are shown in Figures 3, 4 and 5 respectively). These results corroborated our observations concerning the genetic polymorphism among the *P. viridiflava* isolates. Furthermore, our results did not reveal any host-specific clustering pattern for the local or deposited strains, since we observed genetic variability even among strains isolated from the same host plant. However, in the *gyrB* tree (Figure 3), the celery isolates formed a consistent phylogenetic cluster divided in two sub-clusters. It is also noteworthy that four *P. viridiflava* isolates from *A. thaliana* (RT228, KNOX249, KNOX753 and DUD6.3a) were clustered together, forming a separate cluster, which was referred to as clade B by Goss and colleagues [16], while all the local isolates seem to be included to clade A [16]. In this dendrogram, the majority of the local isolates fell into three sub-clusters. The first two sub-clusters contained all the celery isolates (PV271 to PV276; Figure 3 celery groups 1 and 2), while the third sub-cluster contained the eggplant (PV3005, PV3006), acanthus (PV570, PV574a) and the artichoke (PV608, PV609) isolates. However, the local isolates from tomato (TKK615, PV441, PV442), blite (PV527) and melon (PV612) were not included in any of the three abovementioned sub-clusters. These local isolates were closely linked to the *P. viridiflava* reference strains that originated from bean (PDDCC2848 and CFBP2107), *A. thaliana* (SL243.1b, SL2501b), *Cerastium vulgatum* (ME751.1a), *Draba verna* (ME753.1a) and *Cardamine parviflora* (ME756.1a) (Figure 2). Nevertheless, the topology of the celery isolates in this dendrogram follows the topology described for the local isolates (Figure S3A).

Similarly, another dendrogram was created utilizing the *rpoD* gene sequences deposited in GenBank (Figure 4). However, the *rpoD* sequences deposited in GenBank were considerably fewer than those for *gyrB*. In the *rpoD* tree, almost all the local *P. viridiflava* isolates were scattered yet forming three main groups. The first group contained half of the local isolates (local cluster) including all the tomato, eggplant, acanthus and artichoke isolates, while the two others hosted the local celery isolates (celery groups 1 and 2). However, two of the local isolates, (PV527 from blite and PV612 from melon) were grouped separately from all the other local isolates. The blite isolate was grouped with strain BC2506 originating from *Brassica napus*, while the melon isolate was grouped with strains originating from bean and *Ribes aureum* (BFBP2107, PDDCC2848 and NCPPB 963 respectively).

The dendrogram constructed from the *rpoB* sequences deposited in GenBank (Figure 5) revealed even less information due to the lack of deposited sequences. Nevertheless, findings from this analysis appear to be similar to those derived from the analysis of *gyrB* and *rpoD*. No host-specific clustering pattern emerged, even in the case of celery isolates. Although these isolates were grouped again in two groups, they did not appear to be closely linked. In this *rpoB*-derived phylogeny, some of the local isolates appear closely linked yet had a scattered pattern and were separated from the rest of the isolates. However, this may be merely an artifact due to the small number of publicly deposited sequences.

## Discussion

*P. viridiflava* is distinguished from many other plant pathogens in being able to infect a large variety of host species including the model plant *A. thaliana* [16]. This presumably reflects a greater degree of hidden genetic variability and is of great interest because it provides a basis to understand how *P. viridiflava* infect different plant/tissues, and could support the development of tools for disease control and management. Also, *P. viridiflava* is often reported as an opportunistic pathogen [13] and thus could experience selection pressures during the epiphytic phase of its life history that are less prevalent in single-host pathogens [16].

*P. viridiflava* has a broad distribution following no particular geographic map structure. Furthermore, variation among *P. viridiflava* isolates from specific hosts appears to be equivalent to the variation among isolates from different hosts, at least for most of the hosts [16]. Goss and colleagues suggested that *P. viridiflava* is not adapted specifically to any host plant species at the local level, since the genetic variation observed within *Arabidopsis* isolates follows the genetic variation also observed in a global sample of isolates from different hosts. This stands in contrast to studies with related plant pathogenic bacteria, which generally show either little variation [36] or high levels of geographically structured variation [37]. Furthermore, a worldwide sample of *P. syringae* pv. *tomato* and *P. syringae* pv. *maculicola* showed unique fingerprints for almost all isolates [38].

In this study, we examined the patterns of genetic variation among *P. viridiflava* isolates collected from various host plants growing in various areas of the island of Crete (South Greece). Although there is a growing interest in elucidating the population structure and genetic variation in many plant pathogenic bacteria, there is limited data available in the case of *P. viridiflava*. The genetic polymorphism of *P. viridiflava* isolates from Crete was determined by rep-PCR (BOX and ERIC) as well as by the partial *gyrB*, *rpoD* and *rpoB* gene sequencings, and phenotypic profiling by pathogenicity and biochemical tests. The pathogenicity screens and biochemical profiling did not reveal any polymorphism among the isolates examined and thus did not enable us to further study the genetic variability of the local *P. viridiflava* isolates.

However, the ability of rep-PCR for snapshotting the whole bacterial genomes makes it ideal for intraspecific population analyses as previously described for other species [29], [39]. Furthermore, the sequencing of specific genomic fragments was employed for further investigation of the population variability. Although analysis of 16S rDNA sequence is frequently used, the degree of resolution obtained is not sufficient to reveal the real intraspecific relationships because of the extremely slow rate of rDNA evolution [1]. As previously reported, the 16S rDNA-based phylogeny, derived from a single gene, does not necessarily represent the phylogeny of the organisms [40]. Thus, we chose to develop phylogenies based on three housekeeping genes, *gyrB*, *rpoD* and *rpoB,* which have been shown to be useful in grouping isolated strains of several bacterial species and has been extensively used previously [41], [42]. The *gyrB, rpoD* and *rpoB* partial sequences in combination with the results obtained from the rep-PCR amplification enabled us to investigate the diversity in the populations of *P. viridiflava*. Because these genes evolved much faster than 16S rDNA, they provide higher resolution in dendrogram generation [43].

Our analysis revealed that the *gyrB* phylogenetic tree for the local isolates was topologically almost identical to the tree based on the rep-PCR fingerprinting, while the phylogenetic tree based on the *rpoB* and *rpoD* gene sequences revealed clearly different patterns of variation (Figures 2A and S3). It is noteworthy that the *gyrB* sequence used for our analysis includes 36 parsimony informative positions (a site is parsimony-informative if it contains at least two types of nucleotides, and at least two of them occur with a minimum frequency of two), while the *rpoD* and *rpoB* sequences had 27 and 14 respectively. This indicates that the *gyrB* gene sequence is more informative for phylogenetic studies and intra-species genetic variability of *P. viridiflava*, a fact that has also been stated previously for other bacterial species [1].

However, as previously reported [16], the combination of several individual sequence fragments in phylogenetic tree generation results in significant alterations in the associations among isolates compared to those in the trees derived for the individual loci. In our case, the phylogenetic trees obtained from the combined *gyrB*, *rpoD* and *rpoB* sequences showed substantial loss in substructure compared to trees generated for the individual loci. As proposed by Goss et al. [16], this observation may be suggestive of different recombination activities for each locus taking place in *P. viridiflava* isolates (Figure S3). Strikingly, in all three cases the local celery isolates clustered distinctly and separately from the other local isolates. This observation contrasts with the view that *P. viridiflava* is not adapted to host plant species at the local level [16].

Even though the phytopathological and the biochemical profiling of the celery isolates were identical to the rest of the isolates examined, the differentiation seen in the molecular characterization led us to examine these isolates against a broader range of *P. viridiflava* strains by including *gyrB*, *rpoD* and *rpoB* sequences deposited in GenBank (Figures 3, 4, 5 and S3A). Our analysis revealed a very interesting pattern in which almost all the local isolates were grouped together and in separate clusters from the other isolates deposited in GenBank. This independent grouping of the Cretan isolates has been described previously for other plant pathogens [44], [45]. The island of Crete, located in the south-central Mediterranean basin, constitutes an isolated terrestrial land part between three continents; Europe, Africa and Asia. Previous, reports suggested that plant pathogens within the bounds of Greek islands presented separated clades in the generated dendrograms, revealing a remarkable genome polymorphism compared to mainland Europe pathogen populations in which geographic correlations could not be established [44], [45]. Thus, populations from different Greek islands were differentiated from each other, while genetic divergence was also found among subpopulations of the same plot. On the other hand, populations from mainland regions of Greece had high genotypic diversity. This indicates independent evolution of microorganisms in isolated geographic regions like Crete, as appears to be the case with the local *P. viridiflava* isolates.

However, in the *gyrB* phylogenetic tree, the tomato isolates as well as the blite and melon isolates did not group with the other local isolates groups (Figure 3) and the same was observed in the *rpoD* tree for the blite and melon isolates (Figure 4). This possibly indicates a recent arrival of these specific isolates. Unfortunately, we could not obtain consistent results from the *rpoB* tree due to insufficient number of deposited sequences in GenBank. These results indicate that the celery isolates may be adapted to host plant species at the local level.

Finally, our phylogenetic analysis supports the hypothesis that the intra-specific genetic variation of the *P. viridiflava* is not a result of host specific adaptation. However, an exception seen in our study was that of the celery isolates formed a distinct cluster separated from other *P. viridiflava* strains and grouped apart from the other local isolates (Figures 3 and 4). This exemption needs to be further examined by including more geographically distant isolates in order to identify possible host- or geography-related genetic polymorphism. Moreover, the validation of the results against a broader range of samples, coupled with detailed phytopathological (e.g. determination of pathotypes, race resistance, etc.) and molecular attributes may provide a more relevant correlation among molecular and phytopathological traits at the intraspecific level in *P. viridiflava*. This will be critical for obtaining a more comprehensive understanding of the biology, sources and shifts in genetic diversity and evolution of this species and should support the development of molecular identification tools and epidemiological studies in diseases caused by *P. viridiflava*.

## Materials and Methods

### Isolation and identification of bacterial isolates

Affected plant parts, tissues or whole plants were collected and maintained in plastic bags at 6°C until isolations were performed. Samples from infected parts were surface disinfested by placing in 10% ethanol for 30 sec. After two thorough washings in sterile water, small pieces taken from the margin of the infected tissue (leaves or from brown discoloured pith) were ground in a few drops of sterile distilled water. Loopfuls of the suspensions were streaked onto plates of Nutrient Dextrose Agar (NDA) and King's medium B [46]. Plates were incubated for 48 h at 30°C and single colonies were subcultured, checked for purity and stored as slant cultures at 4°C on NDA.

Isolation on King's medium B indicated that the isolated bacteria were fluorescent Pseudomonads. Accordingly, many isolates were initially tested according to the LOPAT tests [7]. Eighteen of these isolates from various hosts (Table 1) were used for further characterization using differential tests presented in Table 2. All methods have been previously described [47].

Each test was repeated at least twice. For further characterization, the following additional tests were performed: Gram stain [48], glucose fermentation in Hugh and Leifson medium [49], and β-glucosidase on arbutin hydrolysis medium [50]. The differential capacity of the isolated *P. viridiflava* strains to fluoresce on iron deficient Misaghi & Grogan's medium [51] containing sucrose, erythritol or DL-lactate as single carbon source, was also tested as described by Jones [22].

### Pathogenicity tests

In preliminary studies all isolates were screened for their ability to induce hypersensitive reaction on tobacco leaves and to cause soft rot of potato slices by previously described methods [47] (Figure S2). The bacterial strains and the host plants used in pathogenicity tests are listed in Table 1. Inoculation methods were performed as previously described with minor changes [13], [15], [47]. All the plants used for inoculations originated from the Department of Plant Sciences of TEI Crete plant collection. They were grown in separate flowerpots (diameter 20 cm) loaded with 3∶1∶1 compost, peat and perlite, respectively, and were inoculated at the 3–5 true leaf stage. Plants were watered with surface drip irrigation. Fertilizer 20-20-20 (N-P-K) was applied weekly by watering. Inoculations were carried out on known host plants and on detached capitulum bracts leaves of artichoke, on immature lemon and pear fruits and on bean pods. Ten plants of each host were cross-inoculated with the strains described in Table S1.

For foliar inoculations on host plants, a suspension of each isolate was sprayed onto leaves of appropriate plants until run off with a hand sprayer. The bacterial inocula were prepared from 24-hrs old King' s medium B plate cultures, suspended in sterile distilled water and adjusted to approximately 10^6^ cfu•ml^−1^ by turbidity measurement with a spectrophotometer at 600 nm and by dilution plate counts. Control plants were sprayed with sterile distilled water.

Stem inoculations were made on tomato and chrysanthemum plants by stabbing with the tip of a sterile toothpick, previously dipped in individual colonies of each strain, into the plant stem just above the second true leaf. Controls were similarly treated with sterile toothpicks. All inoculated plants were held under greenhouse conditions (10–30°C) under intermittent mist (10 sec each hour). Symptoms were evaluated for one month after inoculation.

Cross-inoculation tests were made on detached capitulum bracts leaves of artichoke, on snap bean (*Phaseolus vulgaris* L. cv. Kentucky Wonder) pods and on immature lemon fruits. Surface tissues were swabbed with 70% ethanol and washed in sterile water and stabbed with a sterile needle at six sites. Inoculations were made by deposition of 15 µl of a bacterial suspension adjusted, as above, to approximately10^6^ cfu•ml^−1^. Ten artichoke bract leaves and two immature lemon fruit or bean pods were used for each strain. After inoculation, bracts, fruits and pods were kept in closed transparent plastic boxes lined with moist blotting paper, at room temperature (15–30°C). All inoculations sites were assessed daily for ten days to record disease symptoms.

### Bacterial cultures and genomic DNA preparation

All *Pseudomonas* strains were grown at 26–28°C in King's medium B broth for 24 h. From these cultures, cells were washed with sterile 10 mM MgCl_2_, and a cell suspension was prepared, which was adjusted to an OD_600_ of 0.4, corresponding to 200 cfu•mL^−1^. Aliquots of 500 µL in 2 mL cryo-tubes were stored at −80°C. For DNA extraction, the tube contents were allowed to thaw at room temperature, the cells were lysed for 10 min in a boiling water bath, and the cryo-tubes kept on ice before further use. Total bacterial DNA isolation was carried out using the DNeasy Blood & Tissue Kit from QIAGEN according to the manufacturer instructions.

### Molecular profiling

Detailed characterization of the genetic variability among isolates belonging to *P. viridiflava* species was achieved by DNA fingerprinting, based on BOX-and ERIC-PCR (collectively known as rep-PCR) [23]–[25], as was previously discribed [52]. PCR reaction contained 150 ng template DNA, each of the deoxynucleoside triphosphates at a concentration of 250 nM, primers at a total concentration of 2.5 µM, 2.5 mM MgCl_2_, and 2 units of Taq DNA polymerase (Kapa Biosystems) in a total volume of 20 µl. In the case of BOX-PCR the primer used was the BOXA1R (5′-CTA CGG CAA GGC GAC GCT GAC G-3′) while in the case of ERIC-PCR the primer pair was the ERIC1R/ERIC2 (5′-ATG TAA GCT CCT GGG GAT TCA C-3′ and 5′-AAG TAA GTG ACT GGG GTG AGC G-3′ respectively). The PCR reactions were performed in an Eppendorf Mastercycler Gradient according to the following program: 1 cycle at 95°C for 7 min, 30 cycles consisting of 1 min at 95°C, 30 sec at 53°C and 5 min at 72°C, and 1 cycle at 72°C for 15 min.

In both fingerprinting methods, the patterns were normalized and scorings were performed twice by two independent persons and the results obtained have no impact on the generated tree. The profiles of the rep-PCR gels were transformed into numerical data by P (band presence) and A (band absence) in order to be used for phylogenetic tree construction. Pairwise similarities between electrophoretic patterns were calculated with the Jaccard coefficient and clustering was carried out by the Unweighted Pair Group Method with Arithmetic mean (UPGMA), as previously described [34]. Phylogenetic analyses were conducted in MEGA (Molecular Evolutionary Genetics Analysis) version 5.0 software tool [53].

### PCR amplification and sequencing of *gyrB*, *rpoB* and *rpoD*

PCR amplification of parts of the *gyrB* and *rpoD* genes was carried out following the method and primers described previously [1], [54]. PCR reactions contained 150 ng of template DNA, each of the deoxynucleoside triphosphates at a concentration of 250 µM, total primers at a concentration of 2.5 µM, and 2 units of Taq DNA polymerase (Kapa Biosystems) in a total volume of 20 µl. PCR amplification was performed as follows: initial DNA denaturation at 94°C for 5 min, 35 cycles consisting of 1 min at 94°C, 1 min at 57°C and 2 min at 72°C, and a final step of 72°C for 10 min. Amplified products were electrophoresed on 1.5% agarose gels and purified using QIAquick columns (Qiagen) following the manufacturer's instructions. The nucleotide sequences of *gyrB* and *rpoD* genes were determined directly from the PCR fragments with the reading of the respective PCR amplicons in both directions, using the primer pair UP-1E/APrU (5′- CAG GAA ACA GCT ATG ACC AYG SNG GNG GNA ART TYR A-3′ and 5′- TGT AAA ACG ACG GCC AGT GCN GGR TCY TTY TCY TGR CA-3′ respectively) for *gyrB* gene and PvRpoD1/PvRpoD2 for *rpoD* gene (TGA AGG CGA RAT CGA AAT CGC CAA and 5′-YGC MGW CAG CTT YTG CTG GCA-3′). The sequences were further analysed with MEGA5 software [53].

### Data analysis

Partial sequences of the three housekeeping genes, *gyrB*, *rpoD* and *rpoB*, were obtained from eighteen *P. viridiflava* strains (Table 1). Phylogenetic analysis was carried out using the partial sequences obtained plus corresponding sequences retrieved from NCBI GenBank. Sequence alignment was carried out using the program CLUSTALW [55] and corrected manually.

Phylogenetic trees were established using the UPGMA method [33] as in the dendrogram of Figure 2 or the Neighbour-Joining method [35] as in the dendrogram of Figures 3 and 4. The percentage of replicate trees in which the associated strains clustered together in the bootstrap test (1500 replicates; [56]) was estimated and is shown next to the tree branches. The trees were drawn to scale, with branch lengths in the same units as those of the evolutionary distances used to infer the phylogenetic trees. The evolutionary distances were computed using the Maximum Composite Likelihood method [57] and are in the units of the number of base substitutions per site. All positions containing gaps and missing data were eliminated from the dataset (complete deletion option). Phylogenetic analyses were conducted in MEGA5 [53].

As a measure of goodness of fit for cluster analysis the cophenetic correlation was used [58]. It derives from the comparison of the cophenetic value matrix against the matrix used for the generation of the clustering for 99 permutations. Firstly, the MEGA5 software was used for the estimation of pairwise Genetic Distances among all investigated genotypes. The generated pairwise matrix, regarded as the similarity matrix, was inserted into the SAHN module of NTSYSpc [50] for the generation of the UPGMA tree file and the COPH module of NTSYSpc for the generation of the cophenetic (ultrametric) value matrix. The 2 matrices were inserted into the MXCOMP module of NTSYSpc for the Mantel test. If r≥0.9 the fit is interpreted as very good while an *r* value between 0.8 and 0.9 is interpreted as good fit.

## Supporting Information

Figure S1 *P. viridiflava* natural infections revealing leaf spots on eggplant seedlings (**A**), pith necrosis on tomato plants (**B**), leaf spots on celery (**C**) and bract leaves of artichoke (**D**).
(DOC)

Figure S2 *P. viridiflava* isolates from different hosts did not produce deep black necrotic pit on detached immature lemon fruits (**A**), but caused rust-coloured lesions within 48 h on excised snap bean pods (**B**), had pectinolytic activity (**C**) and induced hypersensitive response on tobacco leaves (**D**).
(DOC)

Figure S3 Phylogenetic trees of the local *P. viridiflava* isolates. The construction of the dendrograms was based on **A:** *gyrB* gene sequence, **B:** *rpoD* gene sequence and **C:** *rpoB* gene sequence. The evolutionary history was inferred using the UPGMA method. The bootstrap consensus tree inferred from 1500 replicates is taken to represent the evolutionary history of the taxa analyzed. Branches corresponding to partitions reproduced in less than 50% bootstrap replicates are collapsed. The percentage of replicate trees in which the associated taxa clustered together in the bootstrap test is shown next to the branches. The tree is drawn to scale, with branch lengths in the same units as those of the evolutionary distances used to infer the phylogenetic tree. The evolutionary distances were computed using the Maximum Composite Likelihood method and are in the units of the number of base substitutions per site. All positions containing gaps and missing data were eliminated. Evolutionary analyses were conducted in MEGA5.
(DOC)

Table S1 Cross inoculation assays of *Pseudomonas* spp. and *Pseudomonas viridiflava* local isolates and reference strain. **+**: Compatible reaction. **−**: Incompatible reaction.
(DOC)

Table S2 Comparison of *P. viridiflava* local isolates from different hosts found in the island of Crete and other fluorescent *Pseudomonas* species used in differential nutritional and biochemical tests. (+) = positive; (−) = negative; NT = not available.
(DOC)

Table S3 Bacterial strains obtained from GenBank used for *gyrB*, *rpoD* and *rpoB* phylogenetic analysis.
(DOC)
