# Supplementary material for: Pseudomonas viridiflava, a Multi Host Plant Pathogen with Significant Genetic Variation at the Molecular Level
Source: PLoS One. 2012 Apr 27;7(4):e36090. doi: 10.1371/journal.pone.0036090 (PMC3338640; doi:10.1371/journal.pone.0036090)
Supplement: Figure S1 — P. viridiflava natural infections revealing leaf spots on eggplant seedlings (A), pith necrosis on tomato plants (B), leaf spots on celery (C) and bract leaves of artichoke (D). (DOC) [file pone.0036090.s001.doc]

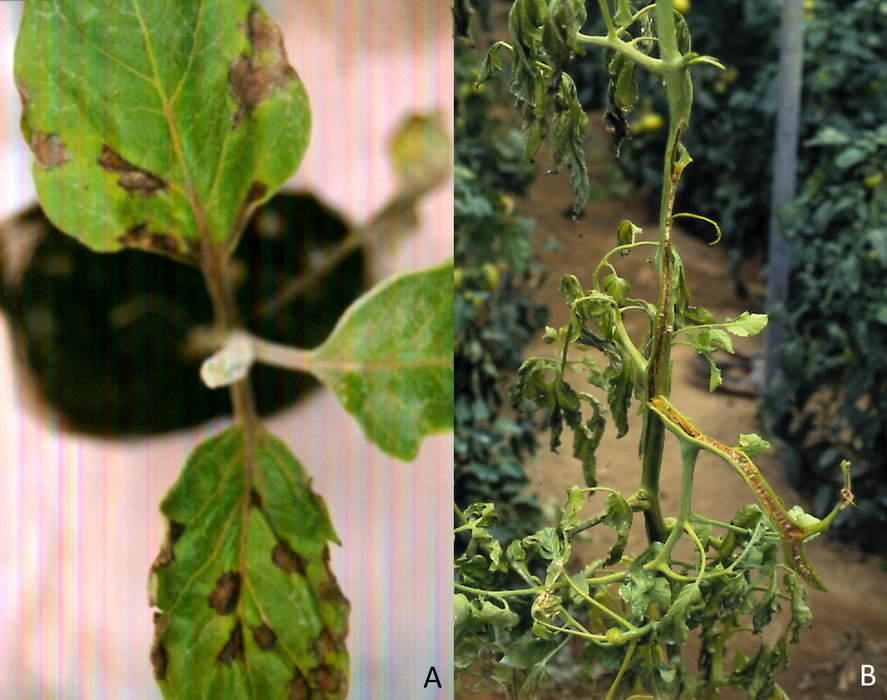

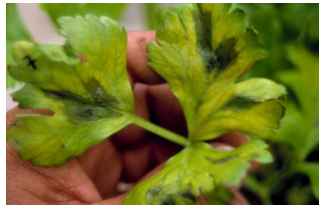


C


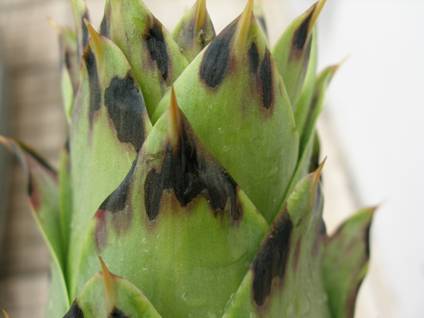


D

**Supplementary Figure S1**: *P. viridiflava* natural infections revealing leaf spots on eggplant seedlings (**A**), pith necrosis on tomato plants (**B**), leaf spots on celery (**C**) and leaf spots on bract leaves of artichoke (**D**).
