# Supplementary material for: Pseudomonas viridiflava, a Multi Host Plant Pathogen with Significant Genetic Variation at the Molecular Level
Source: PLoS One. 2012 Apr 27;7(4):e36090. doi: 10.1371/journal.pone.0036090 (PMC3338640; doi:10.1371/journal.pone.0036090)
Supplement: Figure S2 — P. viridiflava isolates from different hosts did not produce deep black necrotic pit on detached immature lemon fruits (A), but caused rust-coloured lesions within 48 h on excised snap bean pods (B), had pectinolytic activity (C) and induced hypersensitive response on tobacco leaves (D). (DOC) [file pone.0036090.s002.doc]

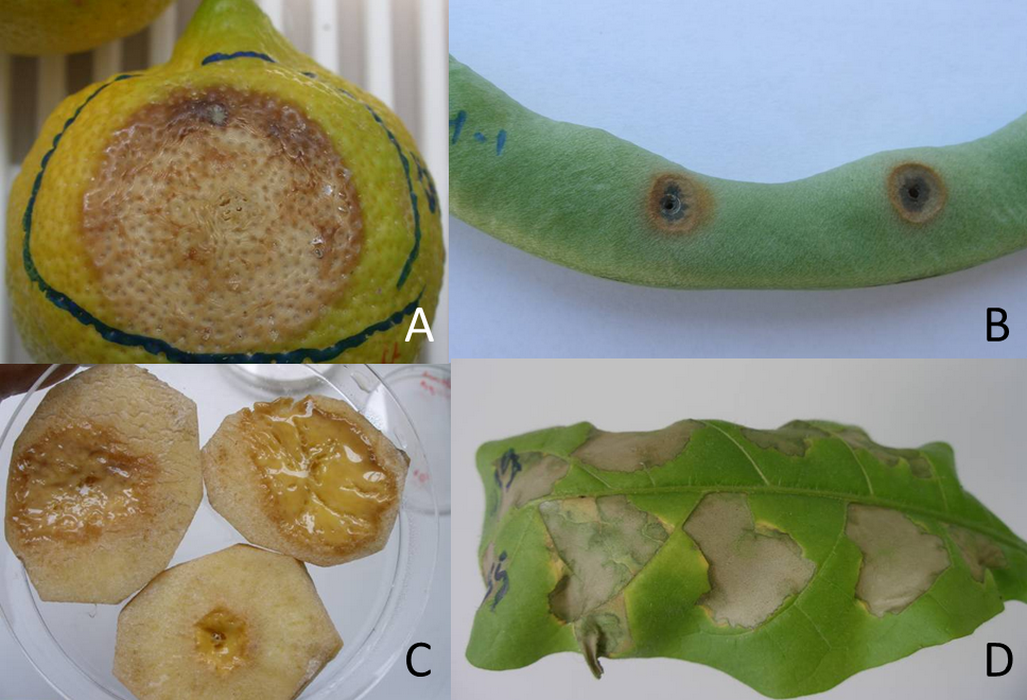


**Supplementary Figure 2:** *P. viridiflava* isolates from different hosts, did not produce the deep black necrotic pit symptoms on detached lemon fruits (**A**), caused rust-coloured lesions within 48 h on excised snap bean pods (**B**), revealed pectinolytic activity on slices of potato tubes (**C**) and induced hypersensitive response on tobacco leaves (**D**).
