# Supplementary material for: Pseudomonas viridiflava, a Multi Host Plant Pathogen with Significant Genetic Variation at the Molecular Level
Source: PLoS One. 2012 Apr 27;7(4):e36090. doi: 10.1371/journal.pone.0036090 (PMC3338640; doi:10.1371/journal.pone.0036090)
Supplement: Figure S3 — Phylogenetic trees of the local P. viridiflava isolates. The construction of the dendrograms was based on A: gyrB gene sequence, B: rpoD gene sequence and C: rpoB gene sequence. The evolutionary history was inferred using the UPGMA method. The bootstrap consensus tree inferred from 1500 replicates is taken to represent the evolutionary history of the taxa analyzed. Branches corresponding to partitions reproduced in less than 50% bootstrap replicates are collapsed. The percentage of replicate trees in which the associated taxa clustered together in the bootstrap test is shown next to the branches. The tree is drawn to scale, with branch lengths in the same units as those of the evolutionary distances used to infer the phylogenetic tree. The evolutionary distances were computed using the Maximum Composite Likelihood method and are in the units of the number of base substitutions per site. All positions containing gaps and missing data were eliminated. Evolutionary analyses were conducted in MEGA5. (DOC) [file pone.0036090.s003.doc]

**
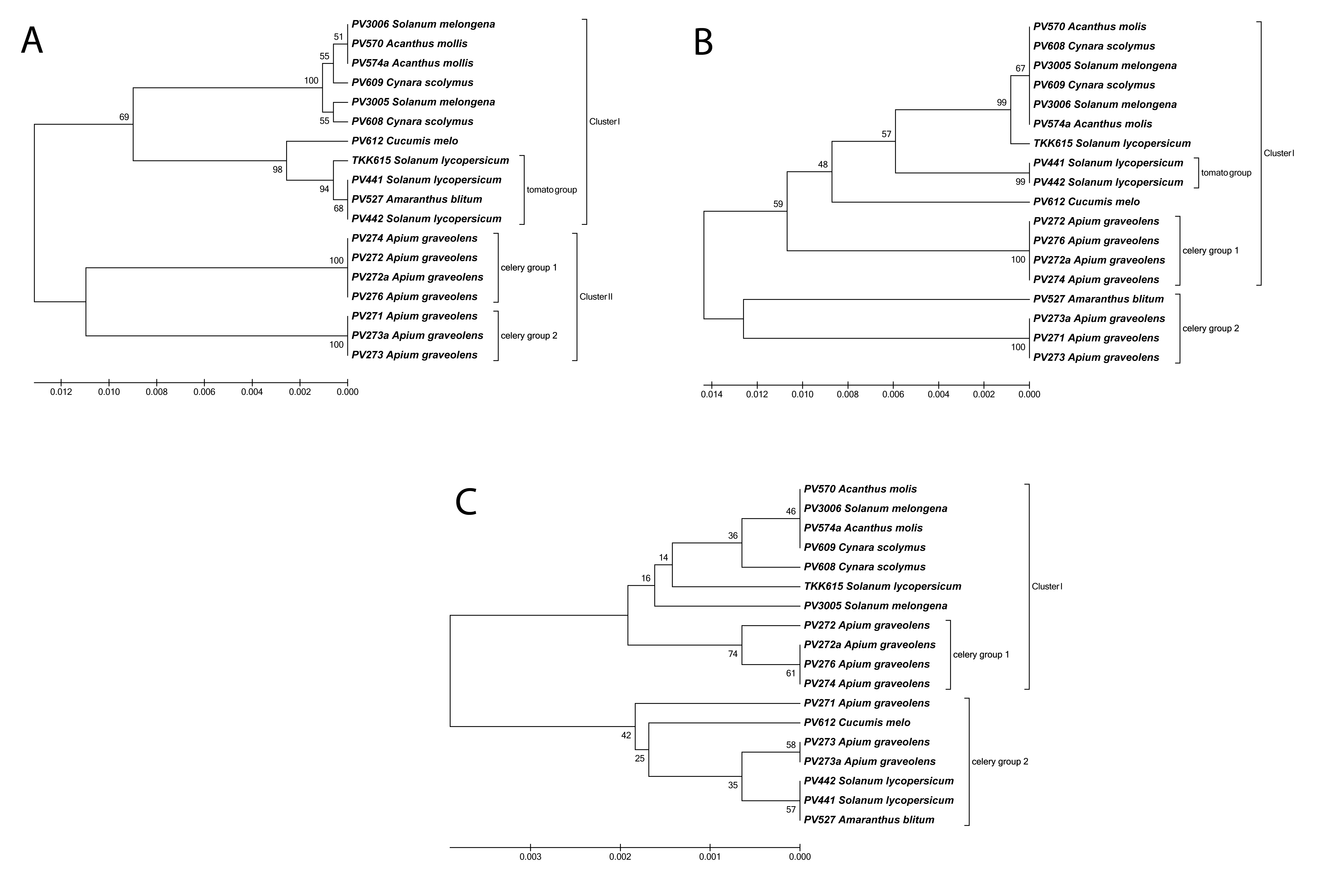
**

**Supplementary Figure 3:** Phylogenetic trees of the local *Pseudomonas viridiflava* isolates. The construction of the dendrograms was based on **A:** *gyrB* gene sequence, **B:** *rpoD* gene sequence and **C:** *rpoB* gene sequence. The evolutionary history was inferred using the UPGMA method. The bootstrap consensus tree inferred from 1500 replicates is taken to represent the evolutionary history of the taxa analyzed. Branches corresponding to partitions reproduced in less than 50% bootstrap replicates are collapsed. The percentage of replicate trees in which the associated taxa clustered together in the bootstrap test (1500 replicates) is shown next to the branches. The tree is drawn to scale, with branch lengths in the same units as those of the evolutionary distances used to infer the phylogenetic tree. The evolutionary distances were computed using the Maximum Composite Likelihood method and are in the units of the number of base substitutions per site. The analysis involved 18 nucleotide sequences. All positions containing gaps and missing data were eliminated. Evolutionary analyses were conducted in MEGA5.
