# Supplementary material for: Pseudomonas viridiflava, a Multi Host Plant Pathogen with Significant Genetic Variation at the Molecular Level
Source: PLoS One. 2012 Apr 27;7(4):e36090. doi: 10.1371/journal.pone.0036090 (PMC3338640; doi:10.1371/journal.pone.0036090)
Supplement: Table S1 — Cross inoculation assays of Pseudomonas spp. and Pseudomonas viridiflava local isolates and reference strain. +: Compatible reaction. −: Incompatible reaction. (DOC) [file pone.0036090.s004.doc]

|  | **Plant species used in cross inoculations** | | | | | | |
| --- | --- | --- | --- | --- | --- | --- | --- |
| ***Pseudomonas* species:** | *Solanum lycopersicum* var. ACE | *Solanum melongena* | *Apium graveolens* | *Amaranthus blitum* | *Cynara scolymus* var. *Lardati* | *Cucumis melo* | *Chrysanthemum morifolium* |
| *Lycopersicon esculentum* isolates: TKK615, PV441, PV442 | + | + | + | + | + | + | + |
| *Solanum melongena* isolates: PV3005, PV3006 | + | + | + | + | + | + | + |
| *Apium graveolens* isolates: PV271, PV272, PV272a, PV273, PV273a, PV274, PV276 | + | + | + | + | + | + | + |
| *Amaranthus blitum* isolate: PV527 | + | + | + | + | + | + | + |
| *Acanthus mollis* isolates: PV570, PV574a | + | + | + | + | + | + | + |
| *Cynara scolymus* isolates: PV608, PV609 | + | + | + | + | + | + | + |
| *Cucumis melo* isolate: PV612 | + | + | + | + | + | + | + |
| *P. viridiflava NCPPB1249* | + | + | + | + | + | + | + |
| *P. syringae* pv. *tomato* Pst1 | + | - | - | - | - | - | - |
| *P. syringae* pv. *lachrymans*  Psl110 | - | - | - | - | - | + | - |
| *P. syringae* pv. *syringae*  NCPPB2778 | - | - | - | - | - | - | - |

**Supplementary Table 1**: Cross inoculation assays of *Pseudomonas* spp. and *Pseudomonas viridiflava* local isolates and reference strain

**+**: Compatible reaction

**-**: Incompatible reaction
