# Supplementary material for: Pseudomonas viridiflava, a Multi Host Plant Pathogen with Significant Genetic Variation at the Molecular Level
Source: PLoS One. 2012 Apr 27;7(4):e36090. doi: 10.1371/journal.pone.0036090 (PMC3338640; doi:10.1371/journal.pone.0036090)
Supplement: Table S2 — Comparison of P. viridiflava local isolates from different hosts found in the island of Crete and other fluorescent Pseudomonas species used in differential nutritional and biochemical tests. (+) = positive; (−) = negative; NT = not available. (DOC) [file pone.0036090.s005.doc]

**Table S2:** Comparison of *P. viridiflava* local isolates from different hosts found in the island of Crete and other fluorescent *Pseudomonas* species used in differential nutritional and biochemical tests. (+) = positive; (-) = negative; NT = not available

|  | ***Solanum lycopersicum*** | ***Solanum melongena*** | ***Apium graveolens*** | ***Amaranthus blitum*** | ***Cynara scolymus* & *Acanthus mollis*** | ***Cucumis melo*** | ***P. viridiflava* NCPPB1249** | **Other *Pseudomonas* species** | | | |
| --- | --- | --- | --- | --- | --- | --- | --- | --- | --- | --- | --- |
|  |  |  |  |  |  |  |  | *syringae* pv. *lachrymans* | *savastanoi* pv. *savastanoi* | *syringae* pv. *syringae* | *syringae* pv. *tomato* |
| **Use for growth** |  |  |  |  |  |  |  |  |  |  |  |
| D(-) Mannitol | + | + | + | + | + | + | + | + | + | + | + |
| D(+) Cellobiose | - | - | - | - | - | - | - | - | - | - | - |
| D(-) Sorbitol | + | + | + | + | + | + | + | + | + | + | + |
| D(+) Trehalose | - | - | - | - | - | - | - | - | - | - | - |
| D(+) Sucrose | - | - | - | - | - | - | - | + | + | + | + |
| i-Inositol | + | + | + | + | + | + | + | + | + | + | + |
| L(-) Rhamnose | - | - | - | - | - | - | - | - | - | - | - |
| D(-) Arabinose | - | - | - | - | - | - | - | - | - | - | - |
| Adonitol | - | - | - | - | - | - | - | - | - | - | - |
| Betaine | + | + | + | + | + | + | + | + | + | + | + |
| Erythritol | + | + | + | + | + | + | + | + | - | + | + |
| DL-Lactate | + | + | + | + | + | + | + | + | + | + | + |
| L(-) Lactate | + | + | + | + | + | + | + | - | + | + | - |
| L(+) Tartrate | **+** | - | - | - | - | - | - | - | - | - | - |
| D(-) Tartrate | + | + | + | + | + | + | + | - | - | - | + |
| Malonate | + | + | + | + | + | + | + | + | + | + | + |
| Anthranilate | - | - | - | - | - | - | - | - | - | - | - |
| L-Valine | - | - | - | - | - | - | - | - | - | - | - |
| β-Alanine | - | - | - | - | - | - | - | - | - | - | - |
