# Supplementary material for: Pseudomonas viridiflava, a Multi Host Plant Pathogen with Significant Genetic Variation at the Molecular Level
Source: PLoS One. 2012 Apr 27;7(4):e36090. doi: 10.1371/journal.pone.0036090 (PMC3338640; doi:10.1371/journal.pone.0036090)
Supplement: Table S3 — Bacterial strains obtained from GenBank used for gyrB, rpoD and rpoB phylogenetic analysis. (DOC) [file pone.0036090.s006.doc]

**Table S3:** Bacterial strains obtained from GenBank used for *gyrB*, *rpoD* and *rpoB* phylogenetic analysis.

| **Strain No.** | **Host** | ***gyrB* GenBank No.** | ***rpoD* GenBank No.** | ***rpoB* GenBank No.** | **Origin** |
| --- | --- | --- | --- | --- | --- |
| LU5.1a | *Arabidopsis thaliana* | AY606746.1 | N/A | N/A | [16] |
| SP3.1a | *Arabidopsis thaliana* | AY606798.1 | N/A | N/A | [16] |
| ME210.1b | *Arabidopsis thaliana* | AY606748.1 | N/A | N/A | [16] |
| BC2509 | *Brassica napus* | HM190238.1 | HM190246.1 | N/A | [59] |
| PT210.1a | *Arabidopsis thaliana* | AY606762.1 | N/A | N/A | [16] |
| LU13.1a | *Arabidopsis thaliana* | AY606744.1 | N/A | N/A | [16] |
| LP23.1a | *Arabidopsis thaliana* | AY606743.1 | N/A | N/A | [16] |
| LU19.1a | *Arabidopsis thaliana* | AY606745.1 | N/A | N/A | [16] |
| BC2496 | *Brassica napus* | HM190233.1 | HM190241.1 | N/A | [59] |
| PNA3.3a | *Arabidopsis thaliana* | AY606760.1 | N/A | N/A | [16] |
| BC2508 | *Brassica napus* | HM190237.1 | HM190245.1 | N/A | [59] |
| BC2495 | *Brassica napus* | HM190232.1 | HM190240.1 | N/A | [59] |
| BC2497 | *Brassica napus* | HM190234 | HM190242.1 | N/A | [59] |
| BC2506 | *Brassica napus* | HM190235.1 | HM190243.1 | N/A | [59] |
| RM225.1b | *Arabidopsis thaliana* | AY606770.1 | N/A | N/A | [16] |
| LH215.1b | *Arabidopsis thaliana* | AY606739.1 | N/A | N/A | [16] |
| ME758.1a | *Lamium purpureum* | AY606759.1 | N/A | N/A | [16] |
| ME754.1a | *Stellaria media* | AY606756.1 | N/A | N/A | [16] |
| KNOX752.1a | Unidentified host | AY606730.1 | N/A | N/A | [16] |
| ME755.1a | *Veronica* sp. | AY606757.1 | N/A | N/A | [16] |
| LH204.1a | *Arabidopsis thaliana* | AY606737.1 | N/A | N/A | [16] |
| RT751.1a | Unidentified host | AY606788.1 | N/A | N/A | [16] |
| RM757.1a | *Veronica* sp. | AY606776.1 | N/A | N/A | [16] |
| RM754.1a | *Stellaria media* | AY606774.1 | N/A | N/A | [16] |
| RM755.1a | *Lepidium campestre* | AY606775.1 | N/A | N/A | [16] |
| BOR4.2a | *Arabidopsis thaliana* | AY606715.1 | N/A | N/A | [16] |
| SL250.1b | *Arabidopsis thaliana* | AY606794.1 | N/A | N/A | [16] |
| ME756.1a | *Cardamine parviflora* | AY606758.1 | N/A | N/A | [16] |
| ME753.1a | *Draba verna* | AY606755.1 | N/A | N/A | [16] |
| ME751.1a | *Cerastium vulgatum* | AY606754.1 | N/A | N/A | [16] |
| SL243.1b | *Arabidopsis thaliana* | AY606793.1 | N/A | N/A | [16] |
| CFBP2107 | *Phaseolus* sp. | HM190239 | HM190247.1 | N/A | [59] |
| PDDCC2848 | *Allium cepa* | AB039427.1 | AB039520.1 | N/A | [1] |
| RT228.1b | *Arabidopsis thaliana* | AY606786.1 | N/A | N/A | [16] |
| KNOX753.1a | Unidentified host | AY606731 | N/A | N/A | [16] |
| KNOX249.1b | *Arabidopsis thaliana* | AY606728.1 | N/A | N/A | [16] |
| DUD6.3a | *Arabidopsis thaliana* | AY606721.1 | N/A | N/A | [16] |
| NCPPB963 | *Ribes aureum* | N/A | FN433264.1 | N/A | [54] |
| BC2507 | *Brassica napus* | N/A | HM190244.1 | N/A | [59] |
| ATCC 13223T | Unidentified host | N/A | N/A | FN554764 | [42] |
| LMG2354 | Unidentified host | N/A | N/A | AJ748184 | [19] |
| LMG12647 | Unidentified host | N/A | N/A | AJ748183 | [19] |
| LMG2352T | Unidentified host | N/A | N/A | AJ717483 | [19] |
| *P. syringae* pv. *ribicola* CFBP2348 | Unidentified host | N/A | N/A | AJ973033 | [19] |
| *Pseudomonas* sp. R-32840R | Unidentified host | N/A | N/A | AM944720 | [52] |
| *Pseudomonas syringae* pv. *primulae* CFBP1660 | Unidentified host | N/A | N/A | AJ973034 | [19] |
| *Pseudomonas* *cichorii* LMG2162T | Unidentified host | N/A | N/A | AJ717418 | [19] |
| *Pseudomonas* sp. R-33145 | Unidentified host | N/A | N/A | AM944723 | [52] |
